# Supplementary material for: Dilemmas in Elderly Diabetes and Clinical Practice Involving Traditional Chinese Medicine
Source: Pharmaceuticals (Basel). 2024 Jul 16;17(7):953. doi: 10.3390/ph17070953 (PMC11279884; doi:10.3390/ph17070953)
Supplement: Supplementary file 1 [file pharmaceuticals-17-00953-s001.zip › Additional File S1- Search strategy and selection criteria.pdf]

Search strategy and selection criteria

We searched PubMed and the Cochrane Central Register of Controlled Trials (CENTRAL) for relevant publications of meta-analysis and systematic reviews using combinations of Medical Subject Headings (MeSHs), free text terms and Boolean operators. Searches were limited to work published in English or Chinese. We sought additional publications from the reference lists of identified papers, and from the libraries of authors. Where possible, the research cited in this series was from overviews and network meta-analysis published in the previous 5 years, with the exception of key research publications. The literature search was done on February 20, 2024. Meta-analysis and systematic reviews on non-human (such as animals) were excluded.

Also, trials registered on the ClinicalTrials.gov (https://clinicaltrials.gov/), WHO International Clinical TrialsRegistry PlatformSearch Portal (https://trialsearch.who.int/), Chinese Clinical Trial Registry (ChiCTR, https://www.chictr.org.cn/), and International Traditional Medicine Clinical Trial Registry (ITMCTR, http://itmctr.ccebtcm.org.cn/zh-CN) was also searched as supplementary materials. Trials withdrawn were ignored.

Example of search strategy in Pubmed:

#1 Prevent*[Title/Abstract]

#2 (Diabetes[Title]) OR (Diabetes mellitus[Title])

#3 #1 AND #2

#4 (Impaired glucose tolerance[Title]) OR (Prediabetes[Title]) OR (Pre-diabetes[Title])

#5 #3 OR #4

#6 (Traditional Chinese Medicine) OR (Chinese herbal) OR (Zhong Yi Xue) OR (Chinese Drugs, Plant) OR (Chinese Plant Extracts) OR (Bioactive Ingredients) OR (Formula) OR (Formulations) OR (decoction) OR (natural product*)

#7 #5 AND #6
