# Supplementary material for: Dilemmas in Elderly Diabetes and Clinical Practice Involving Traditional Chinese Medicine
Source: Pharmaceuticals (Basel). 2024 Jul 16;17(7):953. doi: 10.3390/ph17070953 (PMC11279884; doi:10.3390/ph17070953)
Supplement: Supplementary file 1 [file pharmaceuticals-17-00953-s001.zip › Additional File S2-Table S1-S3.pdf]

**Table S1. Registered trials on TCM and natural products for primary prevention of diabetes**

| Main ID | Year | Country | Condition/ disease | Invention | Status |
|---|---|---|---|---|---|
| ChiCTR-PRC-09000401 | 2009 | China | Prediabetes | Jinqi Jiangtang Tablet | Completed |
| ACTRN12612000128897 | 2012 | China | Prediabetes | Chinese herbal medicine Jiangtang Xiaozhi | Completed |
| ChiCTR-TRC-12001936 | 2012 | China | Prediabetes | Xiaotang seven-herb formula | Completed |
| CTRI/2012/05/002678 | 2012 | India | Prediabetes | Herbal supplements (SR-L-01, SR-B-01 and SI-S-01) | Completed |
| ChiCTR-TRC-13004048 | 2013 | China | Prediabetes | Ba Duan Jin | Completed |
| CTRI/2013/06/003784 | 2013 | India | Prediabetes | Herbal Siddha formulation Avirai Kudineer | Not yet recruiting |
| CTRI/2013/07/003812 | 2013 | India | Prediabetes | Herbal product containing mixture of plant extracts | Recruiting |
| NCT02146157 | 2014 | United States | Prediabetes | Herb and Mineral Combination Product | Completed |
| CTRI/2015/01/005471 | 2015 | India | Prediabetes | Ayurvedic Polyherbal formulation | Completed |
| NCT01964703 | 2015 | Korea | Prediabetes | Rubus Occidentalis Extract | Completed |
| NCT02848053 | 2016 | China | Non-diabetes | Tianqi Capsule | Not yet recruiting |
| ChiCTR-INR-16008891 | 2016 | China | Prediabetes | Acupuncture and herbal medicine intervention combines with lifestyle intervention | Pending |
| ChiCTR-IPR-16008107 | 2016 | Hong Kong, China | Prediabetes | Planta-scraping therapy | Recruiting |
| NCT02706821 | 2016 | Korea | Prediabetes | Persimmon Leaf Extract | Completed |
| NCT03029390 | 2016 | Mexican | Prediabetes | Berberine Versus Metformin | Active, not recruiting |
| ChiCTR1800016549 | 2018 | China | Prediabetes | TCM comprehensive scheme of prevention and treatment | Pending |
| ChiCTR1800019413 | 2018 | China | Prediabetes | Tangzhifang | Pending |
| ChiCTR1800019450 | 2018 | China | Prediabetes | Seabuckthorn puree | Completed |
| IRCT20181022041407N2 | 2018 | Iran | Prediabetes | Curcumin | Pending |
| ISRCTN15680985 | 2018 | India | Prediabetes | Clovinol (clove extract) | Completed |
| NCT03455049 | 2018 | Indonesia | Prediabetes | Andrographis Paniculata | Completed |

| | | | | | |
|---|---|---|---|---|---|
| ChiCTR1900020632 | 2019 | China | Prediabetes | Puer tea | Completed |
| ChiCTR1900020674 | 2019 | China | Prediabetes | Hua Tan Qu Shi Decoction | Pending |
| ChiCTR1900026108 | 2019 | China | Prediabetes | Ba Duan Jin | Recruiting |
| CTRI/2019/02/017455 | 2019 | India | Prediabetes | Ayurvedic drug | Not yet recruiting |
| ITMCTR1900002298/ ChiCTR1900022736 | 2019 | China | Prediabetes | Health education, general life style intervention plus Six Junzi Tang granules | Pending |
| ITMCTR1900002375/ ChiCTR1900023541 | 2019 | China | Prediabetes | IGT Chinese medicine intervention based on APP management | Pending |
| NCT03794232 | 2019 | China | Prediabetes | Fructose Extracted from Jerusalem Artichoke | Completed |
| NCT03917784 | 2019 | Mexican | Prediabetes | Curcumin | Recruiting |
| ChiCTR2000034668 | 2020 | China | Prediabetes | Fucoidin intervention | Pending |
| ChiCTR2000039049 | 2020 | China | Prediabetes | Yi-Jin-Jing combined with Resistance exercise | Recruiting |
| ITMCTR2000003411/ ChiCTR2000034000 | 2020 | China | Prediabetes | Zhenyuan capsule | Pending |
| ITMCTR2000003482/ ChiCTR2000034490 | 2020 | China | Prediabetes | Ba Duan Jin | Recruiting |
| ITMCTR2000003517/ ChiCTR2000035022 | 2020 | China | Prediabetes | Zuo's Acupuncture Treatment | Pending |
| ITMCTR2000004167/ ChiCTR2000040978 | 2020 | China | Prediabetes | Acupuncture and moxibustion | Recruiting |
| KCT0004639 | 2020 | Korea | Prediabetes | Korean red ginseng extracts | Completed |
| NCT04342624 | 2020 | United States | Prediabetes | Cinnamon Spice | Completed |
| ChiCTR2100044474 | 2021 | China | Prediabetes | Food grade Mulberry leaf extract (Reducose) | Completed |
| ChiCTR2100044953 | 2021 | China | Prediabetes | Sodium alginate | Recruiting |
| ChiCTR2100052596 | 2021 | China | Prediabetes | Rubus suavissimus S. Lee | Recruiting |

| | | | | | |
|---|---|---|---|---|---|
| CTRI/2021/02/031315 | 2021 | India | Prediabetes | VAMANA (BIO-PURIFICATORY PROCEDURE OF AYURVEDA) | Not yet recruiting |
| ITMCTR2100004790/ ChiCTR2100045879 | 2021 | China | Prediabetes | Bigu intervention | Recruiting |
| NCT04734132 | 2021 | Spain | Prediabetes | Moringa Oleifera Leaves | Completed |
| NCT04864860 | 2021 | Saudi Arabia | Prediabetes | Seaweed (Ecklonia Cava Extract) | Recruiting |
| NCT05126251 | 2021 | China | Prediabetes | Tangningtongluo Tablet | Not yet recruiting |
| NCT05292300 | 2021 | Turkey | Prediabetes | Lyophilized Dried Cornus Mas L. | Completed |
| TCTR20210401004 | 2021 | Thailand | Prediabetes | Gynostemma pentaphyllum extract tea | Not yet recruiting |
| TCTR20210714004 | 2021 | Thailand | Prediabetes | Snow mushroom | Recruiting |
| TCTR20210816001 | 2021 | Indonesia | Prediabetes | Aloe Vera Juice and Fun Dance Intervention | Recruiting |
| ACTRN12622001108707 | 2022 | New Zealand | Prediabetes | Beetroot compounds and blackcurrant anthocynanins | Recruiting |
| ChiCTR2200060997 | 2022 | China | Prediabetes | Morus alba L. alkaloids (SZ-A) +lifestyle intervention | Completed |
| ChiCTR2200065089 | 2022 | China | Prediabetes | Yinling dampness expell Granules+lifestyle intervention | Pending |
| CTRI/2022/01/039395 | 2022 | India | Prediabetes | Ayurvedic approach | Not yet recruiting |
| IRCT20221220056876N1 | 2022 | Iran | Prediabetes | red beetroot | Recruiting |
| ITMCTR2200006100/ ChiCTR2200060813 | 2022 | China | Prediabetes | Huoxue-Jiangtang Decoction | Pending |
| ChiCTR2300067451 | 2023 | China | Prediabetes | Mobile app based Baduanjin exercise | Pending |
| ChiCTR2300070215 | 2023 | China | Prediabetes | TCM fasting therapy | Pending |
| ChiCTR2300071867 | 2023 | China | Prediabetes | "Clearing/Lowering" method | Completed |
| ChiCTR2300073747 | 2023 | China | Prediabetes | Melbine+ astragalus root extract+health education | Pending |
| ChiCTR2300075569 | 2023 | China | Prediabetes | Kaiyu Jiangzhuo Tongzhi prescription | Pending |
| ChiCTR2300079112 | 2023 | China | Prediabetes | Auricular point sticking and pressing | Recruiting |
| ChiCTR2300079245 | 2023 | China | Prediabetes | Mind-Body Stance | Recruiting |

| | | | | | |
|---|---|---|---|---|---|
| CTRI/2023/11/060192 | 2023 | India | Prediabetes | Masura yusha | Not yet recruiting |
| CTRI/2023/11/060199 | 2023 | India | Prediabetes | Comprehensive Ayurveda intervention | Not yet recruiting |
| ITMCTR2023000057 | 2023 | China | Prediabetes | Thirst granule | Recruiting |
| NCT05749874 | 2023 | China | Prediabetes | Berberine | Not yet recruiting |

**Table S2. Registered trials on TCM and natural products for diabetic cognitive decline.**

| Main ID | Year | Country | Condition/disease | Invention | Status |
|---|---|---|---|---|---|
| IRCT201411112394N14 | 2015 | Iran | T2DM | Resveratrol | Complete |
| ITMCTR2000004084/ ChiCTR2000040268 | 2020 | China | Diabetic Cognitive Dysfunction | Electroacupuncture of Adjust "Zang-fu and Arouse Spirit" | Not recruiting |
| NCT04416841 | 2020 | China | Mild Cognitive Impairment in T2DM | Tai Chi Chuan | Completed |
| ISRCTN15172592 | 2020 | Mexico | Older adults with T2DM | Resveratrol | Recruiting |
| CTRI/2020/01/022963 | 2020 | India | Memory Decline in T2DM | Herbal Drug Saraswat Choorna | Not yet recruiting |
| ITMCTR2000003443/ ChiCTR2000034310 | 2020 | China | T2DM-mild cognitive impairment | Deficiency-excess syndrome TCM | Pending |
| ITMCTR2200006528/ ChiCTR2200062907 | 2022 | China | DM-mild Cognitive Impairment | Acupuncture | Not recruiting |
| ITMCTR2200005496/ ChiCTR2200055242 | 2022 | China | Cognitive impairment of diabetic encephalopathy | Buyang Huanwu Decoction | Recruiting |
| ChiCTR2200063771 | 2022 | China | Cognitive impairment in T2DM | Huayu Tongluo moxibustion | Not recruiting |

Abbreviations: DM= diabetes mellitus; T2DM= type 2 diabetes mellitus

**Table S3. Registered trials on TCM and natural products for diabetic sarcopenia, frailty, and obesity in the elderly.**

| Main ID | Year | Country | Condition/disease | Invention | Status |
|---|---|---|---|---|---|
| NCT02810041 | 2014 | France | Weight in Overweight Subjects VITALIM Senior | Yoghurts Enriched With XXS (Mixture of Natural Polyphenolic Compounds and Plant Extracts) | Completed |
| KCT0008149 | 2021 | Korea | Obesity in Korean Middle-aged women | Auricular Acupressure | Completed |
| NCT05133778 | 2021 | Netherlands | Healthy and overweight middle-aged adults | Sweet Orange and Pomegranate Extract Supplementation | Recruiting |
| ChiCTR2300072775 | 2022 | China | Obesity in middle-aged and elderly people | acupoint catgut embedding which based on sihai and qijie theroy | Recruiting |
| TCTR20230516001 | 2023 | Thailand | Overweight and obesity in elderly | germinated black hang rice extract | Completed |
| RBR-8df2h4 | 2018 | Brazil | Elderly people with loss of muscle mass | Acupuncture | Data analysis completed |
| ITMCTR1900002777/ ChiCTR1900027709 | 2019 | China | Elderly patients with Senile myopenia | Baduanjin Exercise | Pending |
| NCT04535336 | 2020 | Taiwan, China | Older Adults With Sarcopenia | Vitality Acupunch Exercise | Completed |
| NCT05145036 | 2021 | China | Elders With Sarcopenia | Tai Chi Exercise | Completed |
| ChiCTR2100051871 | 2021 | China | Sarcopenia in the elderly | Baduanjin intervention | Pending |
| ChiCTR2100051482 | 2021 | China | Elderly with sarcopenia | 'liver detoxifying spleen, kidney nourishing spleen" Wuqinxi exercise | Pending |
| ISRCTN48485253 | 2021 | Mexico | Musculoskeletal Diseases in older adults and prevent sarcopenia | tai chi training | Recruiting |
| ChiCTR2200063921 | 2022 | China | Elderly patients with sarcopenia | Taijiquan intervention | Pending |
| ACTRN12622001058763 | 2022 | New Zealand | Sarcopenia in Older Adults | Curcumin-Fortified Whey Protein Beverage and Strength Training | Not yet recruiting |

| | | | | | |
|---|---|---|---|---|---|
| ChiCTR2300079294 | 2023 | China | Sarcopenia in the Elderly | Electroacupuncture | Recruiting |
| ChiCTR2300074159 | 2023 | China | Sarcopenia among the elderly | TCM, exercise, and nutrition health education | Recruiting |
| NCT05869383 | 2023 | Indonesia | Elderly Patients With Sarcopenia | Ophiochepalus Striatus Extract | Completed |
| ACTRN12623000046606 | 2023 | Malaysia | Older Adults with Sarcopenia | Cosmos caudatus Extract Supplementation | Not yet recruiting |
| ChiCTR2300067356 | 2023 | China | Sarcopenia in the elderly | TCM for tonifying kidney and spleen combined with resistance exercise | Recruiting |
| NCT06289439 | 2024 | Spain | Sarcopenia in Older Adult Women | Green Tea Supplementation | Recruiting |
| NCT01126723 | 2010 | United States | Frailty in Elderly Adults | Tai Chi | Completed |
| JPRN-UMIN000033107 | 2017 | Japan | Frailty and sarcopenia in older adults | Ninjinyoeito | Follow-up completed |
| NCT03675724 | 2018 | United States | Frailty in Older Adults | Fisetin | Recruiting |
| JPRN-UMIN000033107 | 2018 | Japan | Frailty and sarcopenia in older adults | Ninjinyoeito | follow-up completed |
| JPRN-UMIN000031859 | 2018 | Japan | Frailty in aged patients | Kampo treatment | Recruiting |
| NCT03430037 | 2018 | United States | Frailty in Older Adults | Fisetin | Recruiting |
| ChiCTR2000040875 | 2020 | China | Elderly frailty | meridian beating | Recruiting |
| ChiCTR2100050857 | 2021 | China | Older Adults with Cognitive Frailty | Baduanjin Exercise | Recruiting |
| ChiCTR2100042851 | 2021 | China | Community older adults with cognitive Frailty | Mindfulness-based Tai Chi Chuan (MTCC) | Completed |
| NCT04549103 | 2021 | Singapore | Pre-Frail/Frail Older Adults | Baduanjin | Completed |
| ITMCTR2200006786 | 2022 | China | Pre-frail elderly with immunosenescence | standardized Taichi intervention | Pending |
| NCT05629728 | 2022 | Korea | Frail Older Adults | Tai Chi | Recruiting |
| ChiCTR2300073905 | 2023 | China | Frail elderly people | Taijiquan | Recruiting |

| | | | | | |
|---|---|---|---|---|---|
| ChiCTR2300070053 | 2023 | China | Senile weakness | Baduanjin | Pending |
| ChiCTR2300069201 | 2023 | China | Frail older adults | Baduanjin training and vitamin D supplementation | Pending |
| IRCT20230225057526N1 | 2023 | Iran | Frail Older Adults | Foot Reflexology | Recruiting |
| ACTRN12623000046606 | 2023 | Malaysia | Older Adults with Muscle Loss | Cosmos caudatus Extract Supplementation | Not yet recruiting |
